# The Relation of the Inhalation Anesthetics to Modern Dental Surgery

**Published:** 1918-08

**Authors:** Harry R. Trick

**Affiliations:** Buffalo, New York. Instructor in Oral Surgery, Dental Department; Instructor in General Surgery, Medical Department, University of Buffalo. Assistant Attending Surgeon, Buffalo General Hospital


### The Relation of the Inhalation Anesthetics to Modern Dental Surgery.

By HARRY R. TRICK, M. D., F. A. C. S.,

Buffalo, New York.

Instructor in Oral Surgery, Dental Department; Instructor in General Surgery, Medical Department, University of Buffalo. Assistant Attending Surgeon, Buffalo General Hospital.

It is very difficult, if not quite impossible, for the present-day surgeon to visualize the handicaps under which those worked who practiced in the pre-anesthetic days.

Our occasional experience of performing some slight operation without an anesthetic will give us a faint idea of the harrowing experiences they were frequently obliged to undergo and from which there seemed to be no escape.

A thoughtful consideration of their predicament should excite little wonder that they generally attempted to evade what many times must have seemed to be their real duty, and instead to perform hurried, incomplete and sacrificial operations as compared to the deliberate, thorough, and conservative procedures of today.

The wonder is that they were able to do so well as they did!

We all can bear witness to the statement that of all the pains to which flesh is heir, none is more unnerving than the pain incident to some acute dental pathology and the attempt to combat or remove this pathology without the aid of some anesthetic causes a shock to the patient that may be difficult to overcome and a loss of nervous energy in the surgeon that frequently repeated would lead to exhaustion.

It must have been because of the relatively frequent repetition of such experiences that the hope was kept constantly in their minds that some day some pain-killing substance would be produced, but their experiences did not prompt many of them to make any very serious attempt to discover it.

Even if we make all manner of generous excuses for them we must admit that their faith was too child-like, if not censurable, because—"We must all remember that the thought of painless surgery did not burst upon the world **de novo** in the year 1846 when Morton gave the first public demonstration of ether anesthesia. Running through all medical history, there is a constant reference to the abolishing of pain, and even among the ancients many fairly successful attempts were made.''

Viewed from our standpoint, another inexplicable oversight was the fact that for a number of years prior to 1846 it had been the custom to give what were known as ''ether frolics'' as part of an evening's entertainment and to exhibit the effects of laughing-gas on the stage and in halls of learning.

It frequently happened that during these exhibitions some of the participants would be severely bruised without apparently being aware of the injury and this fact was noted and commented upon, but it remained for Horace Wells, a dentist of Hartford, Conn., to apply these facts to his professional needs.

Unfortunately, his attempted demonstration in the Massachusetts General Hospital was a failure, probably due to faulty technique, and the general use of nitrous-oxide in surgery was thereby considerably delayed.

Morton, however, worked along different lines. He seems to have been of a crafty nature. He was familiar with the work of Wells, in fact, they had been at one time partners, but he was not satisfied with the effects of nitrous-oxide as then administered, so he continued a search for a more satisfactory agent.

He received the suggestion to use sulphuric ether from Dr. Chas. T. Jackson, a prominent and versatile physician of Boston, who acted as preceptor to Morton when he matriculated in Harvard Medical School.

Morton immediately tried out the effect of ether on himself and sundry patients. He also experimented with a variety of masks for its administration and continued to receive valuable advice and information from Dr. Jackson but all these various endeavors he carried out furtively, until the time of the public demonstration at the Massachusetts General Hospital, October 16, 1846. Even then he attempted to make it appear that he had discovered a new and mysterious drug by disguising the odor with aromatic oils and naming the combination ''letheon.''

The effect of the announcement of the success of the demonstration was electrical and was the direct cause of the subsequent exhibition of the most sordid greed by all the parties named.

Horace Wells and E. E. Marcy of Hartford had also been using ether at this time but considered it too dangerous, although, they did not discontinue its use until it had been suggested to Valentine Mott who referred to it in a paper published in the Boston Medical and Surgical Journal in 1845.

It also appears that Dr. Crawford Long, a practicing physician of Jefferson, Jackson County, Georgia, had used ether in surgical practice even as early as 1842, but made no formal report of his experience until so late that he just prevented Morton receiving an appropriation from the Congress of the United States.

In a Commemorative Address delivered at the Medical Department of the University of Buffalo, October 16, 1896, Roswell Park said:

"There probably is every reason to think that, either by accident or design, a condition of greater or less insensibility to pain had been produced between 1820 and 1846, by a number of different people, educated and ignorant, but that no one had the originality or the hardihood to push these investigations to the point of determining the real usefulness of ether. This was partly from ignorance, partly from fear, and partly because of the generally accepted impossibility of producing safe insensibility to pain. So, while independent claims sprang up from various sources, made by aspirants for honors in this direction, it is undoubtedly as properly due to Morton to credit him with the introduction of this agent as an anesthetic as to credit Columbus with the discovery of the New World, in spite of certain evidences that some portions of the American continent had been touched upon by adventurous voyagers before Columbus ever saw it."

Sir James Paget has summed up the relative claims of these four contestants very tersely in an article entitled "Escape from Pain," published in the **Nineteenth Century** for December 1879.

He says:

"While Long waited, and Wells turned back, and Jackson was thinking, and those to whom they had talked were neither acting nor thinking, Morton, the practical man, went to work and worked resolutely. He gave ether successfully in severe surgical operations; he loudly proclaimed his deeds and he compelled mankind to hear him."

Thus, an impartial verdict, rendered long after the heat of the discussion, extends the honor of introducing the inhalation anesthetics for the relief of suffering humanity, to Dr. Wm. T. G. Morton.

That is his reward and in a way it is quite in keeping with the immeasurable debt humanity owes for such a blessing.

It should not be overlooked that the words **anesthetic** and anesthesia were after-thoughts and were suggested by Dr. Oliver Wendell Holmes.

A number of similar substances were promptly put in the field but ether, nitrous-oxide, chloroform, and ethyl chloride still hold the popular confidence.

The effect on the dental profession was staggering. Literally thousands decided to make dentistry their life work. The places for instruction were swamped.

Although there had been for a number of years a few dental colleges in the country they were separate schools and it was not until 1868 that "Harvard did what she ought to have done at the outset. She opened a dental department and began the teaching of dentistry as a branch of medicine, establishing therefore a separate degrée; D. M. D., (Dentariae Medicinae Doctor). In 1874 the University of Michigan established a dental department, and a little later the University of Pennsylvania did the same."

Other schools followed rapidly. The Dental Department of the University of Buffalo was established in 1892, at which time there were between forty and fifty dental colleges in the United States, most of which owed their origin quite directly to the increased demand for instruction above noted.

This enormously increased field of usefulness imposed certain obligations, such as better preparation for the study of dentistry and an improved and extended curriculum, all of which, I am proud to say have been met in a very satisfactory way.

Although the present high place in medicine that dentistry occupies can be credited to the inhalation anesthetics, their use in dentistry has been largely reserved for the more formidable procedures since the introduction of cocaine by Koller at the Congress of German Oculists in Heidelberg in 1884.

Under these circumstances, when the decision rests on an inhalation anesthetic, the respnosibility should be divided with a trained anesthetist and he should decide on the particular anesthetic to be used and the method of its administration.

In any instance the method of administration should be the simplest possible to meet the indications.

To my mind simplicity of administration has a salutary psychic effect that cannot be ignored and the psychic side of anesthesia is a most important one.

BIBLIOGRAPHY:

Gwathmey—Anesthesia.

Park—Surgical and Scientific Papers.

Mumford—History of Medicine in America.

